# Crosstalk between the Tumor Microenvironment and Immune System in Pancreatic Ductal Adenocarcinoma: Potential Targets for New Therapeutic Approaches

**DOI:** 10.1155/2018/7530619

**Published:** 2018-12-18

**Authors:** Paola Parente, Pietro Parcesepe, Claudia Covelli, Nunzio Olivieri, Andrea Remo, Massimo Pancione, Tiziana Pia Latiano, Paolo Graziano, Evaristo Maiello, Guido Giordano

**Affiliations:** ^1^Fondazione IRCCS Casa Sollievo della Sofferenza, UO di Anatomia Patologica, Viale Cappuccini 1, 71013 San Giovanni Rotondo, FG, Italy; ^2^Department of Diagnostics and Public Health, Section of Pathology, University and Hospital Trust of Verona, P.le L.A. Scuro 10, 37134 Verona, Italy; ^3^Biology Department, University of Naples Federico II, Via Mezzocannone 8, 80134 Naples, Italy; ^4^“Mater Salutis” Hospital, ULSS 9, Via C. Gianella 1, 37045 Legnago, Verona, Italy; ^5^Department of Sciences and Technologies, University of Sannio, Via Port'Arsa 11, 82100 Benevento, Italy; ^6^Fondazione IRCCS Casa Sollievo della Sofferenza, UO di Oncologia Medica, Viale Cappuccini 1, 71013 San Giovanni Rotondo, FG, Italy

## Abstract

Pancreatic ductal adenocarcinoma is a lethal disease for which radical surgery and chemotherapy represent the only curative options for a small proportion of patients. Recently, FOLFIRINOX and nab-paclitaxel plus gemcitabine have improved the survival of metastatic patients but prognosis remains poor. A pancreatic tumor microenvironment is a dynamic milieu of cellular and acellular elements, and it represents one of the major limitations to chemotherapy efficacy. The continued crosstalk between cancer cells and the surrounding microenvironment causes immunosuppression within pancreatic immune infiltrate increasing tumor aggressiveness. Several potential targets have been identified among tumor microenvironment components, and different therapeutic approaches are under investigation. In this article, we provide a qualitative literature review about the crosstalk between the tumor microenvironment components and immune system in pancreatic cancer. Finally, we discuss potential therapeutic strategies targeting the tumor microenvironment and we show the ongoing trials.

## 1. Introduction

Pancreatic ductal adenocarcinoma (PDAC) is an aggressive disease accounting as the fourth leading cause of cancer-related deaths worldwide, and it is estimated to become the second within 2030 [1]. PDAC incidence and mortality are similar, and the five-year survival rate for all stages is around 8% [2]. The primary therapeutic strategies include surgery and chemotherapy. Unfortunately, majority of patients have unresectable, locally advanced, or metastatic disease at the time of diagnosis and treatment is only palliative in this setting [3]. Chemotherapy is the cornerstone of advanced PDAC treatment even if patients' outcome has been disappointing with this approach because of the occurrence of chemoresistance [4]. In addition, target agents have failed to improve survival both alone and in combination with standard chemotherapy [5]. Single-agent gemcitabine has been the mainstay of advanced PDAC treatment since 1997, despite of a small survival benefit [6]. In the last decade, drug portfolio has been enriched by novel combinations like FOLFIRINOX and nab-paclitaxel (nab-P) plus gemcitabine (GEM) that represent the standards of care in metastatic disease management [7, 8]. Nevertheless, treatment effectiveness is limited and patients' prognosis remains very poor. Several factors could explain the reduced efficacy of chemo- and targeted therapies: signalling redundancy, the role of stem cells, the tumor microenvironment (TME), and desmoplastic stroma [9–11]. PDAC is a “milieu” of distinct elements that compose the so-called TME, including fibroinflammatory stroma, extracellular matrix, infiltrating immune cells, and cancer cell population [12, 13]. A growing knowledge of the PDAC pathogenesis has led to better understanding of the immune components' role within the TME. Stimulation and mobilization of the human immune system as well as the enhancement of TME antitumor capacity have become a research focus in PDAC treatment [14, 15]. In this article, we will provide a qualitative literature review about the crosstalk between the TME components and immune system in PDAC. Finally, we will discuss potential therapeutic strategies targeting the TME and we will show the ongoing trials in this field.

## 2. Literature Research Methods

**Figure 1:**
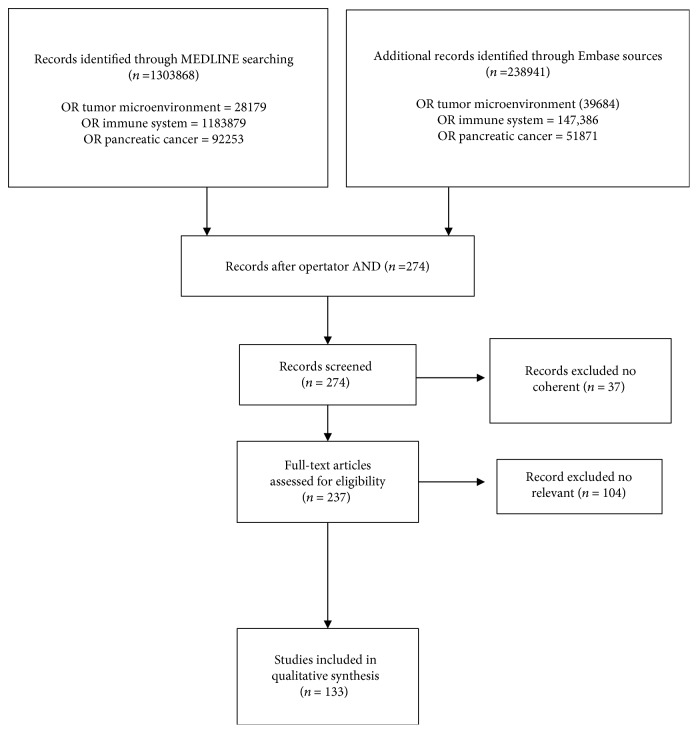
Preferred reporting items for systematic reviews and meta-analysis (PRISMA) protocol used for the systematic review.

A systematic review of the literature was performed in compliance with the PRISMA guidelines [16]. Article titles or full text up to May 2018 using electronic databases MEDLINE and Embase was screened. The primary search terms included “tumor microenvironment,” “immune system,” and “pancreatic cancer” in the article titles using operator “OR.” Later, to narrow the scope of the review, operator “AND” was applied on the extracted records by using the abovementioned terms. Two hundred seventy-four articles met eligibility criteria for our qualitative systematic review. 37 papers were excluded because they were not coherent as well as 104 because they were not relevant, resulting in 133 full texts being included (Figure 1). In addition, ASCO, ASCO GI, and ESMO abstracts published during the last three years were evaluated in order to detect the most recent clinical data about drugs targeting the TME. Trials with negative or not clinically relevant results were excluded from this article. Finally, ClinicalTrials.gov website was interrogated and “recruiting,” “active, not recruiting,” and “not yet recruiting” trials in PDAC were selected. The National Cancer Institute Drug Dictionary was consulted to verify that the mechanism of action of screened drugs was clearly directed against the TME and immune system.

## 3. Pancreatic Cancer and the TME

**Figure 2:**
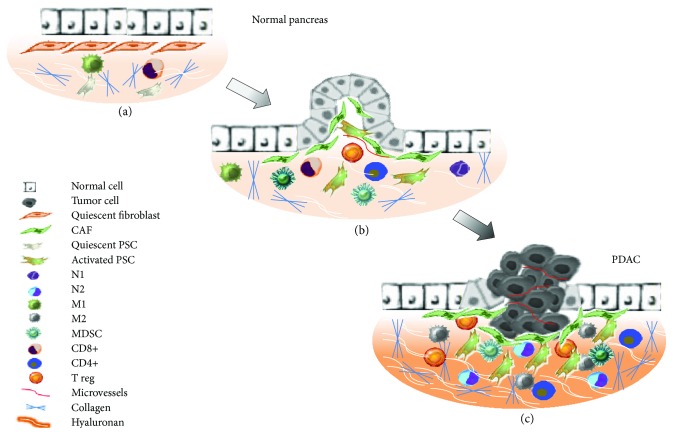
Descriptive model representing pancreatic microenvironment changes during PDAC carcinogenesis. (a) In the normal pancreas, connective tissue, resident fibroblasts (PFs), pancreatic stellate cells (PSCs), immune cells, and vascular cells play a critical role in tissue repair and wound healing. (b) Pancreatic tissue damages and oncogenic mutations lead to carcinogenesis and disrupt normal communications between PSCs/PFs and immune and vascular cells, determining a favorable microenvironment for cancer progression. Soluble and growth factors produced by cancer cells activate PSCs and PFs that play a key role in the development and maintenance of stromal cancer compartment increasing extracellular matrix synthesis. (c) The intense fibrotic stromal reaction of PDAC is characterized by PSCs, PFs, vascular elements, immune cells, and acellular components such as collagens and hyaluronan, fibronectin, cytokines, and growth factors stored in the extracellular matrix. In this stage of disease MDSCs, M2, N2, and T_regs_ induce a protumor phenotype.

A TME is an intricate system with peculiar physical and biochemical features, in which interactions between tumor and stromal cells promote carcinogenesis, progression, metastasis, and therapeutic resistance [17, 18]. Consistently, extracellular matrix (ECM) elements, vascular networks, and lymphatic networks show an abnormal behaviour within the TME [19]. In the normal pancreas, connective tissue, resident fibroblasts (PFs), pancreatic stellate cells (PSCs), immune cells, and vascular cells play a critical role in tissue repair and wound healing (Figure 2(a)). In response to pancreatic tissue damage, injured acinar cells secrete proinflammatory, proangiogenic growth factors and cytokines activating immune cells, PSCs/PFs, and vascular cells to restore normal pancreatic function (Figure 2(b)) [20]. However, in the presence of oncogenic mutations like KRAS, TP53, SMAD4, and CDKN2A, genetically altered epithelial cells transform into cancer cells and disrupt normal communications between PSCs and immune and vascular cells, creating a favorable microenvironment for cancer progression (Figure 2(c)) [21]. PDAC is characterized by a profuse fibrotic stromal reaction called “desmoplasia,” composed of cellular elements such as PSCs, PFs, vascular elements, immune cells, and acellular components such as collagens, fibronectin, cytokines, and growth factors stored in the extracellular matrix (Figure 2(c)). Abundance of stroma is a unique characteristic of PDAC, and it is well demonstrated that the microenvironment influences both responses to treatment and survival of PDAC patients [22]. Notably, during disease progression, tumor stroma exerts pressure on blood vessels, causing their constriction and hypoxic niche formation [23]. Consequently, low-oxygen content in the tumor induces the hypoxia-inducible factor 1 (HIF1A) stabilization. HIF1A mediates activation of different signals that alter metabolic pathways, induce invasiveness, promote chemoresistance, and lead to a poor prognosis of the patient. Upon hypoxic stress, HIF1A accumulates and compensates for low oxygen by increasing glycolysis and glucose uptake in the cells. The consequent metabolic switch from oxidative phosphorylation to aerobic glycolysis results in the production of lactate and acidification of the extracellular environment [24, 25]. Hypoxic conditions, acidic extracellular pH, and high interstitial fluid pressure in the TME are additional drivers of tumorigenesis and tumor progression [17]. The TME also develops an adapted metabolism, in which malignant epithelial cells consume proteins and lipids as a source of energy. Finally, an invasive epithelial to mesenchymal transition (ETM) and a metastatic phenotype complete the PDAC microenvironment [18, 26]. Although desmoplasia represents more than 80% of the tumor mass, the PDAC microenvironment is also replete with immune cells [27]. In particular, PDAC infiltrate is rich of T-cells, also known as tumor-infiltrating lymphocytes (TILs) [28]. Consistently, even if innate and adaptive immune responses are active against the tumor, PDAC by itself induces local and systemic immune dysfunction or immunosuppression to prevent eradication by effector immune cells [29]. Recent studies have showed that PDAC immune cells interact with TME components, resulting in the inactivation of the cytotoxic antitumoral response [29]. In this scenario, the TME could influence treatment efficacy through different mechanisms, including drug delivery modulation, immunosuppression, vascular remodelling, metabolic activities, and signalling pathways involved in DNA repair and apoptosis [30].

## 4. Cellular Component of the TME

**Figure 3:**
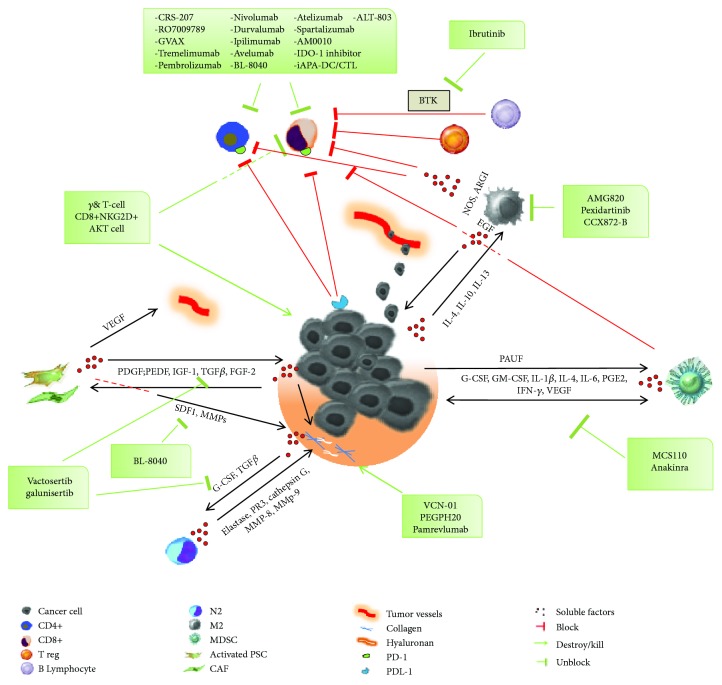
Crosstalk between cancer cells, the TME, the immune system, and potential therapeutic targets. TME components interplay with cancer cells (black arrows) through cytokines and growth factors becoming active and causing tumor proliferation, invasiveness, and metastasization. Communication between PDAC cells and activated PSCs/PFs induces soluble factor secretion increasing ECM production. This continued crosstalk determines immunosuppressive effects on TME immune infiltrate (red lines). Tumor-infiltrating lymphocytes produce high levels of PD-1 and interact with PDL-1 overexpressed by PDAC cells, resulting in T lymphocyte depletion. Several molecules (target agents or immunotherapies) with different mechanisms of action (green boxes) may interfere in the crosstalk between cancer cells and the TME restoring immune response, directly killing tumor cells, or destroying ECM components (green lines).

The cellular component includes pancreatic fibroblasts (PFs), pancreatic stellate cells (PSCs), vascular cells, and inflammatory/immune cells (Figure 2(c)). All these components interact with each other and with cancer cells in a complex fashion (Figure 3) [31]. In normal condition, PFs are inert and spindle-shaped cells in the connective tissue, embedded in physiological ECM. Differently, PDAC cells recruit PFs to the tumor mass and convert them in cancer-associated fibroblasts (CAFs) through genetic and epigenetic changes [32]. CAFs are a characteristic type of myofibroblastic cells expressing alpha-smooth muscle actin (*α*-SMA) that contribute to PDAC progression [32]. In the normal pancreas, quiescent PSCs are located in the periacinar space representing only a small proportion of all pancreatic cells (Figure 2(a)) [33]. Quiescent PSCs have a low mitotic index and synthesize matrix proteins [34]. Following activation by toxins, oxidant stress, smoking, cytokines, and growth factors, quiescent PSCs acquire a myofibroblast-like phenotype and are called “activated PSCs” (Figure 2(c)) [31]. Notably, activated PSCs express *α*-SMA and play a key role in the development and maintenance of the stromal cancer compartment, mediating an extracellular matrix synthesis increase [35, 36]. Microvessels contribute to normal pancreatic microenvironment regulation. Differently, in PDAC, a dysregulated vascular network is demonstrated. In particular, pericytes normally recruited by endothelial cells (ECs) could migrate from vessels and potentially undergo a pericyte-myofibroblast transition within the PDAC microenvironment [37, 38]. Furthermore, ECs could be indirectly activated by CAFs or tumor cells through secretion of proteases in the ECM [39]. Inflammatory and immune cells are crucial elements in the pancreatic TME, and their involvement in generating chemoresistance has become a matter of intense research. Bone marrow-derived cells (BMDCs) are recruited to the pancreatic stroma, leading to early carcinogenesis and metastases together with PSCs, CAFs, and inflammatory cells [40]. BMDCs differentiate into several cell types and contribute to both neovascularization and fibrosis in PDAC stroma by activating PSCs, myeloid-derived suppressor cells (MDSCs), and mast cells (Figure 2(c)) [41, 42]. High levels of MDSCs lead to premetastatic niche formation, tumor invasiveness, angiogenesis stimulation, and worse prognosis [43]. PDAC cells recruit also monocytes from bone marrow within the TME, transforming them into macrophages. Tumor-associated macrophages (TAMs) have been described as promoters of cancer initiation, progression, and metastasization and protect tumors from cytotoxic agents. In particular, TAMs can be converted into M1-like inflammatory macrophages that could activate an immune response against the tumor or into M2-like immunosuppressive macrophages that promote tumor immunity and tumor progression (Figures 2(b) and 2(c)) [44]. M2 TAMs have effect on tumor survival by inhibiting T-cell response and recruiting regulatory T-cells (T_reg_ cells) that negatively influence cytotoxic T-cells [45]. Elevated CD4+ in the TME can promote tumor growth blocking CD8+-related antitumoral response [46]. Recently, several studies showed that B lymphocytes support PDAC carcinogenesis and progression stimulating cancer cell proliferation, suppressing CD8+ cells through the Bruton tyrosine kinase (BTK) pathway [47, 48]. Finally, depending on the stimuli, neutrophils may differentiate into two subtypes in PDAC. N1 neutrophils may potentially kill tumor cells under negative regulation of IFN-*β*. On the other hand, under TGF-*β* and G-CSF stimulation, neutrophils activate into a protumor phenotype called N2 (Figures 2(b) and 2(c)) [49].

## 5. Acellular Component of the TME

The acellular component of the TME is made of collagens I, III, and IV; periostin; fibronectin; and hyaluronic acid (Figure 2(c)) [50, 51]. In many solid tumors as PDAC, elevated collagen deposition contributes to form the stromal barrier influencing both drug resistance and poor prognosis. ECM remodelling is made by lysyl oxidases (LOX), a family of amine oxidases that catalyze the posttranslational crosslinking of collagen molecules, thus favoring biogenesis and maturation. Tumor stroma is characterized by abnormal LOX expression; consequently, high collagen deposition is possible [52]. Hyaluronic acid (HA) is a glycosaminoglycan composed of repeated N-acetyl glucosamine and glucuronic acid units, alternating in *β*-1,3 and *β*-1,4 linkages. HA synthesis is regulated by HA synthases (HAS 1–3) and *α*-SMA-positive myofibroblasts, and its degradation is carried by six hyaluronidases [53, 54]. An elevated HA level has been found in PDAC where it binds and traps water molecules in the ECM, causing high pressure on neighboring structures as well as elevated interstitial fluid pressure within the tumor [53]. Furthermore, it is known that HA binds several receptors as CD44, receptor for hyaluronan-mediated motility (RHAMM), lymphatic vessel endothelial HA receptor-1 (LYVE-1), hyaluronan receptor for endocytosis (HARE), layilin, and Toll-like receptor 4, implicated in tumor migration, invasion, adhesion, and proliferation [55]. Periostin is an osteoblast-specific factor, preferentially expressed in the periosteum functioning as a cell adhesion molecule, and its expression is 42-fold higher in PDAC compared to that in the normal pancreas [31]. Notably, periostin promotes PDAC cell invasiveness, resistance to hypoxia-induced death, and EMT. Fibronectin (Fn) is one of the most abundant ECM proteins and binds to collagen, periostin, fibrillin, and tenascin-C facilitating their assembly and organization [56]. In PDAC, high levels of Fn are secreted by CAFs together with type I and II collagens causing an anisotropic fiber orientation that drives cancer cell migration [57].

## 6. Crosstalk between Cancer Cells, the TME, and the Immune-System in PDAC

The continuous interaction between the glandular neoplastic component and TME has been widely investigated so far. Several authors demonstrated the reciprocal influence of PDAC cells on PSCs via intercellular signalling (Figure 3) [22]. In particular, PDAC cells stimulate PSC activation, proliferation, and migration through cytokines and growth factors such as pigment epithelium-derived factor (PEDF), platelet-derived growth factor (PDGF), PDGF-1, insulin-like growth factor (IGF), and ECM synthesis via TGF-*β* and fibroblast growth factor 2 (FGF2) [20]. On the other hand, PSCs stimulate cancer cell proliferation by production of paracrine factors as TGF-*β*, FGF2, PDGF, and epidermal growth factor (EGF) and inhibit apoptosis [20]. Moreover, metalloproteinase (MMPs) synthesis is mainly correlated to TGF*β*-1 and tumor necrosis factor- (TNF-) *α* [58]. Secretion of MMPs, stroma cell-derived factor-1 (SDF-1), acidic secreted protein and rich in cysteine (SPARC), PDGF, and EGF by PSCs induces invasion and migration (Figure 3). Furthermore, PSCs promote invasion and metastasis by inducing the EMT phenotype in PDAC cells via loss of adhesion intercellular proteins such as E-cadherin and enhance tumor angiogenesis by secretion of vascular endothelial growth factor (VEGF) [59, 60]. Another candidate factor that has received some attention in recent years is the hepatocyte growth factor (HGF), which is secreted by activated PSCs and has a pivotal role in cancer cell proliferation and migration binding its transmembrane cell surface receptor c-MET, which is expressed on cancer cells. Furthermore, c-MET is present on the surface of ECs, enhancing PSC-EC interaction with a potential role in angiogenesis and metastatic spread [61]. Activation of fibroblasts into CAFs is induced by numerous cytokines and growth factors like TGF-*β*, EGF, PDGF, and FGF2 secreted in the TME (Figure 3) [62]. Tumor cells influencing PSCs and CAFs drive ECM remodelling through assembly, alignment, unfolding, and crosslinking of collagen type I and the fibronectin-rich matrix. Interestingly, CAFs produce both signalling factors and exosomes that reinforce the crosstalk with tumor cells [63]. In this context, PDAC cells recruit pericytes via PDGF secretion inducing both chemotaxis from microvessels and pericyte-myofibroblast transition [37]. Furthermore, ECs can be directly induced by cancer cells through soluble factors (FGF-1, FGF-2, VEGFA, and PDGF-B), activation of adhesion receptor (OPG and JAGGED1), gap junctions (CX43), and vesicles (or exosomes) [38]. Contemporarily, BMDCs are attracted in PDAC stroma by growth factors as fibroblast activation protein (FAP), PDGF, TGF-*β*1, VEGF, and EGF produced by tumor cells and participate to PSC activation [40]. In the PDAC microenvironment, cytokines including G-CSF, GM-CSF, IL-1*β*, IL-4, IL-6, prostaglandin E2 (PGE2), IFN-*γ*, and VEGF induce MDSCs to infiltrate the tumor (Figure 3) [64]. MDSCs are myeloid cells that suppress T-cell activation through TGF-*β* secretion, nitric oxide and reactive oxygen species (ROS) production, and arginase-1 depletion. Consistently, cancer cells upregulate a soluble protein named pancreatic adenocarcinoma upregulated factor (PAUF), increasing the accumulation of MDSCs and enhancing their immunosuppressive function (Figure 3) [43]. The intricate crosstalk between PDAC cells and the microenvironment involves also immune elements. Macrophage colony-stimulating factor receptor (M-CSF/M-CSFR) and C-C motif chemokine ligand 2-C-C motif chemokine receptor-2 (CCL2/CCR2) pathways are involved in the recruitment of TAMs. Once within the tumor, TAMs switch towards a M2 phenotype via colony-stimulating factor-1 (CSF-1). M2 are activated by cancer cells through IL-4, IL-10, and IL-13 production and secrete macrophage-derived EGF causing tumor cell migration around blood vessels [65]. Furthermore, M2 release nitric oxide synthase (NOS) and arginase I (ARGI) damaging T lymphocytes through L-arginine depletion in the TME (Figure 3) [66]. Interestingly, neutrophils contribute to tumor growth and invasiveness, producing neutrophil-derived proteases as elastase, PR3, cathepsin G, MMP-8, and MMP-9 that destroy the surrounding ECM [67]. In the dense fibrotic TME, cancer cells activate a wide variety of signalling pathways and suppress both innate and adaptive immune systems by decreasing cytotoxic CD8 T-cells and increasing the presence of immunosuppressive macrophages (M2), neutrophils (N2), and T_reg_ cells (Figure 2(c)) [27]. Otherwise, tumor-infiltrating lymphocytes (TILs) produce high levels of programmed cell death protein 1 (PD-1) and interact with its specific ligand, known as programmed cell death ligand 1 (PDL-1) overexpressed by PDAC cells, resulting in T lymphocyte depletion (Figure 3) [68, 69].

## 7. Clinical Impact of TME and Immune System Components in PDAC

Recently, a wide genome-sequencing programme has been developed in order to better understand PDAC heterogeneity and get information that could have a clinical significance. In particular, whole genome sequencing and copy number variation analyses performed on 100 tumor samples classified four PDAC subtypes depending on chromosomal structure variation: stable, locally rearranged, scattered, and unstable. Each subtype could predict a different therapeutic responsiveness [21]. Subsequently, integrated genomic analysis of 456 PDACs identified 32 mutated genes that aggregate into 10 pathways (K-Ras, WNT, NOTCH, ROBO/SLIT signalling, G1/S transition, TGF-*β*, SWI-SNF, chromatin modification, DNA repair, and RNA processing). Notably, the TGF-*β* pathway is mainly involved in TME modelling, regulation, and crosstalk with the immune system. A further analysis of those pathways defined four PDAC subtypes that correlate with histopathological characteristics and have different prognoses: (a) squamous, (b) pancreatic progenitor, (c) immunogenic, and (d) aberrantly differentiated endocrine exocrine (ADEX). Interestingly, the immunogenic subtype is characterized by a predominant B- and T-cell (CD8+, T_reg_) infiltrate as well as cytotoxic T lymphocyte antigen-4 (CTLA4) and PD-1 upregulation [70]. Consistently, PDAC stromal features, immune elements, and their correlation with patients' outcome have been investigated in several research programmes. Knudsen et al. showed that PDAC stroma could be differentiated into three categories called “mature” with dense collagenous stroma and low number of CAFs, “immature” that is highly cellular and collagen poor, and an “intermediate form.” Among those phenotypes, the immature form strongly correlated with worse prognosis. Additionally, poor overall survival was observed in patients with lower stromal volume, high peritumoral T lymphocytes, monocytes/macrophages, CTLA4, and PDL-1 in TME [71]. Immunohistochemistry analysis performed on 88 PDAC samples demonstrated that patients with high-density M2 macrophage infiltration in the stroma had shorter overall survival than those with low M2 infiltration [72]. Furthermore, neutrophil infiltrates have been observed both in the neighborhood of tumor cells and in the stroma and correlated with undifferentiated tumor growth and poor prognosis in 363 pancreatic tumor samples [73]. Coherently, this pathological evidence could partially explain the prognostic significance of the neutrophil to lymphocyte ratio (NLR) value in the peripheral blood of PDAC patients. Several studies both on resected and metastatic PDACs showed that high NLR were related to significantly shorter OS [74, 75]. The impact of TILs on PDAC patients' prognosis is not yet clarified and the available data are not conclusive. The evaluation of TILs on tumor samples in the cohort enrolled in the PDAC adjuvant CONKO 001 study showed a significant correlation between high TIL levels and longer disease-free survival (DFS) and OS [76]. Those results had no confirmation in the Knudsen et al. data in which no correlation between TILs and survival was found [71]. In contrast, in many studies, the high presence of T_reg_ in TME has shown to unfavorably impact the prognosis [77]. The D-1/PDL-1 axis has a well-established role in different neoplasms including PDAC. This pathway regulates the interaction between tumor cell and lymphocytes and their crosstalk with TME [68]. In the last years, several authors attempted to redefine the clinical relevance of PD-1/PDL-1 expression in PDAC, but also, in this field, the road will be long to run. A retrospective analysis of PDL-1 mRNA expression in 453 PDAC samples showed that PDL-1 upregulation was associated with worse DFS and OS. In the same study, PDL-1 upregulation was correlated with biological parameters, showing some degree of T-cell infiltration, signs of antitumor response, and profiles of lymphocyte exhaustion [78]. The PD-1/PDL-1 prognostic value was also evaluated in a group of 145 PDAC surgical samples. Patients with CD8+ and PD-1+, lymphocytes in the stroma had better outcomes compared to patients with low expression, independently from clinic-pathologic parameters like age, tumor site, TNM staging, resection margins, and previous chemotherapy. In this study, a correlation between the PDL-1 status and Bailey's molecular PDAC classification was found. In particular, PDL-1 mRNA was upregulated in the squamous subtype versus each other subtype [79]. The acellular component of the TME has been investigated in order to understand the clinical significance. A recent meta-analysis examined the clinical status and OS of PDAC patients with high HIF-1*α* expression compared to those with low expression. HIF-1*α* was associated with a higher rate of lymph node metastasis and advanced tumor stage. Notably, HIF-1*α* overexpression was significantly correlated with poor OS [24]. Interestingly, another study found negative correlation between survival and extracellular matrix deposition in primary PDACs. Median survival was significantly higher in low-collagen patients compared to high-level ones. Furthermore, low-HA level patients had longer OS than high-HA level patients. This analysis also indicated that extracellular matrix components, such as collagen and HA, are found in high levels in both primary tumors and metastatic lesions [80].

## 8. Potential Targets for Therapeutic Approaches: Insights into Clinical Data

The TME is involved in the lack of responsiveness to chemo- and target therapies favoring a hypoxic environment, causing difficulty in drug access and limiting the immune infiltration. The crosstalk between TME cellular elements and the immune system promotes a clearly immunosuppressive phenotype (Figure 3) [81, 82]. There is an intense research focused on the TME and immune system as therapeutic targets, and potentially, active agents are under investigation (Figure 3 and Tables 1 and 2).

### 8.1. Targeting Tumor Stroma and the Extracellular Matrix

To date, the only drug approved for metastatic PDAC treatment that works against the TME is Nab-P [83]. Nab-P is an innovative molecule obtained by the combination of traditional paclitaxel with nanoparticles of albumin that binds tumor and stromal SPARC enhancing paclitaxel-selective delivery in PDAC cells [84]. The randomized phase III MPACT study showed that combination of Nab-P and GEM significantly increased median OS, progression-free survival (PFS), and response rates versus GEM alone in metastatic PDAC patients [8]. Unfortunately, a post hoc analysis on PDAC samples of the MPACT study failed to show the prognostic and predictive roles of SPARC [85]. Nab-P plus GEM is actually under investigation as the backbone of chemotherapy for novel combinations with immunotherapies or target agents directed against TME (Table 2). In particular, hyaluronidase treatment has been suggested to enhance degradation of HA [86]. Hyaluronidase synergizes with chemotherapy reducing HA levels and intratumoral pressure and increasing drug penetration [31, 46]. Pegvorhyaluronidase alfa (PEGPH20) was made with polyethylene glycol molecules linked to hyaluronidase, prolonging its half-life to >10 h. An open-label randomized phase 2 trial of PEGPH20 + Nab-P/GEM (PAG) versus Nab-P/GEM (AG) in 279 untreated metastatic PDAC patients showed a superior median PFS for the PAG versus AG, only in patients with high intratumoral HA content. Conversely, a modest trend towards better OS was found only in a small subgroup of high-HA tumor patients [87]. Actually, a global randomized phase III study in metastatic PDAC patients with high HA levels detected by immunohistochemistry is evaluating PAG (Table 2). Connective tissue growth factor (CTGF) is a profibrotic mediator that results as abundant in the stroma of PDAC. A human monoclonal antibody against CTGF (Pamrevlumab, FG-3019) was tested with GEM and erlotinib in stage III or IV PDAC [81]. Moreover, the combination of Nab-P + GEM with or without Pamrevlumab has been investigated in a phase I/II randomized study in locally advanced PDAC patients showing an increased resection rate and subsequent longer survival in the triplet arm [88].

### 8.2. Targeting the Immune Microenvironment

In PDAC, the TFG-*β* signalling pathway is involved in tumor progression and it is associated with poor prognosis. TGF-*β* has been related to tumor aggressiveness and invasiveness and to the activation of PSCs, leading to pancreatic desmoplasia. TGF-*β* is also associated to immune cell regulation, migration, and proliferation [89]. Therefore, targeting the TGF-*β* signalling pathway could be a rational therapeutic approach in PDAC [90]. A randomized phase II study assigned 156 patients to receive Galunisertib (anti-TGF-*β*) plus GEM or placebo plus GEM in stage II to stage IV unresectable PDAC. The combination of Galunisertib/GEM resulted in improvement of OS and PFS and a manageable toxicity profile compared to that of placebo/GEM. A major OS benefit was observed for the subgroup of patients with baseline TGF-*β*1 levels ≤ 4224 pg/mL [91]. Another mechanism that target indirectly the TGF-*β* pathway is the inhibition of the renin-angiotensin system with losartan. Fifty locally advanced PDAC patients were enrolled in a phase II study receiving FOLFIRINOX and losartan for a median of 8 cycles. This combination met the criteria for feasibility without severe toxicities, showing 61% of the R0 resection rate [92]. Vactosertib is a potent, highly selective, oral TGFBR1 inhibitor. Twenty-nine PDAC patients were enrolled in a phase I study, and vactosertib was safe and well tolerated [93]. Anti TGF-*β* agents are currently under investigation in clinical trials both in combination with chemotherapy and immunotherapy in PDAC treatment (Tables 1 and 2). Preclinical data showed that vitamin D analog therapy decreased MDSCs and T_regs_, turning PDAC into a more “immune friendly” microenvironment. Preliminary results of a phase II pilot trial of Nivolumab + nab-P + Cisplatin + Paricalcitol + GEM in previously untreated metastatic PDAC patients showed 80% of the objective response rate and median PFS of 8.2 months. This regimen was related to 100% grade 3-4 thrombocytopenia, 50% grade 3-4 anemia, and 20% grade 3 colitis. This trial is still on going and data presented so far regarded only 10 patients (Table 2) [94]. CCR2 inhibition decreases TAMs and T_regs_, increasing CD8+ and CD4+ cells in pancreatic tumors. A clinical trial evaluating CCR2 oral selective inhibitor CCX872-B in combination with FOLFIRINOX in locally advanced or metastatic PDAC showed 29% of OS at 18 months with no safety issues ascribed to CCX872-B use. Better OS was associated with lower peripheral blood monocyte counts at baseline [95]. The BTK pathway has a role in TME modulation. Ibrutinib demonstrated antitumor activity in preclinical PDAC models inhibiting mast cell degranulation, decreasing tumor-associated inflammation and desmoplasia, and enhancing cytotoxic T-cells [48]. A phase II-III trial is evaluating ibrutinib, in combination with Nab-P/GEM versus Nab-P/GEM alone, in 320 metastatic PDAC patients (Table 2). AM0010 is a covalent conjugate of recombinant IL-10 and polyethylene glycol (PEG), with potential antifibrotic, anti-inflammatory, immunomodulating, and antineoplastic activities. Upon subcutaneous administration, AM0010 may activate cell-mediated immunity against cancer cells stimulating CD8+ T-cell differentiation and expansion (Table 1). In a recent phase II trial, PDAC patients progressing on a median of 2 prior therapy were enrolled to AM0010 + FOLFOX resulting in a 15.8% response rate, 78.9% disease control rate, and 10.2-month median OS with good tolerability [96]. A phase III study of AM0010 with FOLFOX compared to FOLFOX alone as second-line therapy in metastatic PDAC patients is ongoing (Table 2). Recently, immune checkpoint inhibitors have been investigated in metastatic PDAC treatment (Table 1). To date, few data from early clinical trials are available. In particular, anti-PD-1 inhibitors have showed a safe toxicity profile but limited activity in combination with standard chemotherapy in “unselected” PDAC patients [94, 97]. Inhibiting the CSF-1/receptor pathway can reduce the intrinsic or acquired resistance to PD-1 inhibitors. Lacnotuzumab, a humanized antibody directed against CSF-1, in combination with Spartalizumab, anti-PD-1 humanized antibody, is under evaluation in a phase Ib/II study, showing good safety results [98].

## 9. Concluding Remarks

Pancreatic cancer management remains a challenge for oncologists despite that new therapeutic options have showed incremental survival advantage. TME and its components are main actors of tumor aggressiveness and treatment resistance. Stromal barrier, intense ECM production, high interstitial fluid pressure, hypoxia, and acidic extracellular pH contribute to make PDAC a chemorefractory tumor. Moreover, the crosstalk between TME and cancer cells causes immunosuppressive condition within PDAC immune infiltrate. Several signals deeply involved in early carcinogenesis, proliferation, invasiveness, and metastasization are activated by growth factors, chemokines, and cytokines released in this milieu. In the absence of predictive biomarkers for response and patient selection, an intriguing therapeutic approach should aim to normalize stroma, interfere in the crosstalk between TME and cancer cells, and restore the antitumoral activity of the immune system. Therefore, novel potential treatment strategies should include chemo/target/immunotherapy combinations or sequences in order to prevent or overcome resistances and improve outcomes.

## Figures and Tables

**Table 1:** Target agents directed against TME or immunotherapies under investigation in PDAC.

Name	Type/structure	Mechanism of action	Effect
ALT-803	Fusion protein	Binds IL-2/IL-15 receptor beta common gamma chain (IL-2R beta gamma) receptor on natural killer (NK) and CD8+	Activation and increase of NK cell memory CD8+ levels
AM0010	Covalent conjugate of recombinant human interleukin-10 (IL-10) and polyethylene glycol (PEG)	Activates cell-mediated immunity against cancer cells by stimulating the CD8+ differentiation and expansion	Potential antifibrotic, anti-inflammatory, immunomodulating, and antineoplastic activities
AMG 820	Fully human monoclonal antibody (IgG2)	Against the colony-stimulating factor-1 (CSF-1 or M-CSF) receptor c-fms (or CSFR1)	Suppresses recruitment and activation of TAMs
Anakinra	Recombinant human nonglycosylated IL-1 receptor antagonist	Blocks IL-1 activity	Inhibition of VEGF, TNF-*α*, and IL-6 cascade resulting in inhibition of tumor angiogenesis
Atezolizumab	Humanized, Fc optimized	Binds to PD-L1, blocking its binding to and activation of PD-1 on activated T-cells	Enhancement of T-cell-mediated immune response and reversal of T-cell inactivation
Avelumab	Human monoclonal antibody (IgG1)	Binds to PD-L1 preventing interaction with PD-1	May restore immune function activation of cytotoxic T lymphocytes
BL-8040	Short peptide	Binds to the chemokine receptor CXCR4, preventing the binding of stromal-derived factor-1 to the CXCR4 receptor	Decreases tumor cell proliferation and migration
CCX872-B	Small molecule	Human C-C chemokine receptor type 2 (CCR2) antagonist	Inhibition of both CCR2 activation and CCR2-mediated signal transduction
CD8 + NKG2D + AKT cell	Cells	Human CD8+ tumor specific engineered to express the natural killer cell-activating receptor group 2D (NKG2D) and the serine/threonine kinase AKT	Potential immunomodulating and antineoplastic activities
CRS-207	Recombinant Listeria-based cancer vaccine expressing human mesothelin	Listeria invades professional phagocytes within the immune system and expresses mesothelin, activating a cytotoxic T lymphocyte response against mesothelin-expressing tumor cells	Potential immunostimulatory and antineoplastic activities
Durvalumab	Fc-optimized monoclonal antibody	Binds to PD-L1 blocking its binding to and activation of PD-1 expressed on activated T-cells	Reverses T-cell inactivation and activates the immune system to exert a cytotoxic T lymphocyte response against PD-L1-expressing tumor cells
Galunisertib	Small molecule	Antagonist of TGF-*β* receptor type 1 (TGFBR1)	Prevents the activation of the TGF-*β*-mediated signalling pathways inhibiting tumor proliferation
GVAX	Allogeneic cancer vaccine composed of lethally irradiated whole melanoma cancer cells that are genetically modified to secrete the immunostimulatory cytokine granulocyte-macrophage colony-stimulating factor	Stimulates the body's immune system against tumor cells	Enhances the activation of dendritic cells, promotes antigen presentation to both B- and T-cells, and increases IL-2-mediated lymphokine-activated killer cell function
iAPA-DC/CTL	A cell-based product composed of dendritic cells (DCs) pulsed with tumor-associated antigens and devoid of the inhibitory effect of antigen presentation attenuators (iAPA) combined with cytotoxic T lymphocytes	Prevents the expression of APA genes and inhibits attenuation of antigen presentation	Potential immunostimulating and antineoplastic activities
Ibrutinib	Small molecule	Binds to and irreversibly inhibits BTK activity	Prevents both B-cell activation and B-cell-mediated signalling leading to growth inhibition of the malignant B-cells overexpressing BTK
IDO-1 inhibitor	Small molecule	Targets and binds to indoleamine 2,3-dioxygenase 1, a cytosolic enzyme responsible for the oxidation of tryptophan into the immunosuppressive metabolite kynurenine	Restores and promotes proliferation and activation of various immune cells and causes a reduction in T_regs_
Ipilimumab	Recombinant human monoclonal antibody (IgG1)	Binds to CTLA4 expressed on T-cells	Inhibits the CTLA4-mediated downregulation of T-cell activation leading to a cytotoxic T lymphocyte-mediated immune response
M7824	Bifunctional fusion protein composed of a monoclonal antibody against PD-L1 fused to the extracellular domain of human TGF-*β* receptor II	“Trap” for all three TGF-*β* isoforms	Suppressed tumor growth and metastasis
MCS110 (Lacnotuzumab)	Humanized monoclonal antibody	Binds to M-CSF and blocks M-CSF-mediated signalling through the M-CSF receptor CD116	Antineoplastic activities
Nivolumab	Fully human monoclonal antibody (IgG4)	Binds PD-1 and blocks its activation by PD-L1	Activation of T-cell immune responses against tumor
Pamrevlumab	Humanized monoclonal antibody	Binds to connective tissue growth factor (CTGF) preventing the binding to the receptor and its subsequent activation	May prevent and reverse fibrosis; prevents tumor cell proliferation in CTGF-expressing tumor cells
PDR 001 (Spartalizumab)	Humanized monoclonal antibody	Directed against the negative immunoregulatory human cell surface receptor programmed death-1	Prevents PD-1-mediated signalling and results in both T-cell activation and the induction of T-cell-mediated immune responses against tumor cells
PEGPH20	Recombinant form of human hyaluronidase	Degrades hyaluronic acid- (HA-) coating tumor cells	Inhibition of tumor cell growth, lowering of the interstitial fluid pressure and allowing better penetration of chemotherapeutic agents into the tumor bed
Pembrolizumab	Humanized monoclonal immunoglobulin antibody (IgG4)	Directed against PD-1	Restores T-cell activation and immune response
Pexidartinib	Small molecule	Binds to and inhibits phosphorylation of stem cell factor receptor (KIT), colony-stimulating factor-1 receptor (CSF1R), and FMS-like tyrosine kinase 3 (FLT3)	Inhibition of tumor cell proliferation and downmodulation of macrophages, osteoclasts, and mast cells
RO7009789	Recombinant monoclonal antibody	Binds to CD40 on a variety of immune cell types	Activation of antigen-presenting cells (APCs), B-cells, and T-cells, resulting in an enhanced immune response
Tremelimumab	Human immunoglobulin monoclonal antibody (IgG2)	Directed CTLA4	A cytotoxic T lymphocyte immune response against cancer cells
Vactosertib	Small molecule	Inhibitor of the serine/threonine kinase TGFBR1 also known as activin receptor-like kinase 5 (ALK5)	Inhibits the activity of TGFBR1 and prevents TGF-*β*/TGFBR1-mediated signalling and suppresses tumor growth
VCN-01	Adenovirus	Replication-competent adenovirus encoding the human glycosylphosphatidylinositol-anchored enzyme PH20 hyaluronidase	Potential antitumor activity
*γδ* T-cell	Cells	Secrete interferon-gamma	Direct killing of tumor cells, activation of cytotoxic T lymphocyte response against tumor cells

**Table 2:** Current clinical trials investigating strategies directed against TME in PDAC.

Study ID	Setting	Study drugs	Phase	Status
NCT02715804	Metastatic PDAC (I line—HA high pts)	Nab-P + GEM ± PEGPH20	III rand	Recruiting
NCT02923921	Metastatic PDAC (II line)	FOLFOX ± AM0010	III rand	Recruiting
NCT02436668	Metastatic (I line)	Nab-P + GEM ± ibrutinib	II-III rand	Active, not recruiting
NCT02030860	Resectable	Nab-P + GEM ± Paricalcitol	II rand	Active, not recruiting
NCT02243371	Advanced	GVAX + CY + CRS-207 ± Nivolumab	II rand	Active, not recruiting
NCT03006302	Metastatic	Epacadostat + Pembrolizumab + CRS-207 ± CY/GVAX	II rand	Recruiting
NCT02648282	Locally advanced	CY, pembrolizumab, GVAX, and SBRT	II	Recruiting
NCT01088789	Resected	Boost vaccinations^∗^ of pancreatic tumor cell vaccine	II	Recruiting
NCT02826486	Metastatic	BL-8040 + Pembrolizumab	II	Active, not recruiting
NCT03432676	Advanced	IDO-1 inhibitor + Epacadostat + Pembrolizumab in PDAC with CIS/HRD	II	Not yet recruiting
NCT02910882	Localized, unresectable	PEGPH20 + GEM + radiotherapy	II	Active, not recruiting
NCT02451982	Resectable	GVAX + CY ± Nivolumab	I-II rand	Recruiting
NCT03193190	Metastatic	Atezolizumab + Cobimetinib or Atezolizumab + PEGPH20 or Atezolizumab + BL-8040	I-II rand	Recruiting
NCT02210559	Locally advanced	GEM + Nab-P ± FG-3019	I-II rand	Active, not recruiting
NCT02311361	Metastatic	Tremelimumab and/or Durvalumab + radiation therapy	I-II	Recruiting
NCT02583477	Metastatic	Durvalumab	I-II	Active, not recruiting
NCT02305186	Resectable	Pembrolizumab	I-II	Recruiting
NCT02077881	Metastatic	IDO Inhibitor + Nab-P + GEM	I-II	Recruiting
NCT02562898	Metastatic	Ibrutinib + Nab-P + GEM	I-II	Active, not recruiting
NCT02529579	Advanced	iAPA-DC/CTL + GEM	I-II	Recruiting
NCT03180437	Resectable/advanced/metastatic	*γδ* T-cell	I-II	Recruiting
NCT02311361	Unresectable	Tremelimumab and/or MEDI4736 + radiation therapy+	I-II	Recruiting
NCT03451773	Advanced	M7824 + GEM	I-II	Recruiting
NCT02713529	Advanced	AMG 820 + Pembrolizumab	I-II	Active, not recruiting
NCT02807844	Metastatic	MCS110 + Spartalizumab	I-II	Recruiting
NCT02929797	Locally advanced	GEM ± CD8 + NKG2D + AKT cell	I rand	Recruiting
NCT03519308	Resectable	Nivolumab + Paricalcitol	I	Recruiting
NCT02559674	Metastatic	ALT-803 + Nab-P + GEM	I	Active, not recruiting
NCT02588443	Resectable	RO7009789 alone or RO7009789 + Nab-P + GEM ➔ RO7009789 + Nab-P + GEM	I	Recruiting
NCT02345408	Advanced	CCX872-B	I	Active, not recruiting
NCT02550327	Advanced	Nab-P + GEM + Cisplatin + Anakinra	I	Recruiting
NCT02930902	Resectable	Pembrolizumab + Paricalcitol ± Nab-P + GEM	I	Recruiting
NCT02868632	Locally advanced	MEDI4736 + SBRT or Tremelimumab + SBRT or MEDI4736 + Tremelimumab + SBRT	I	Recruiting
NCT01473940	Metastatic	Ipilimumab + GEM	I	Active, not recruiting
NCT02777710	Metastatic	Durvalumab + Pexidartinib	I	Recruiting
NCT02345408	Unresectable	CCX872-B	I	Active, not recruiting
NCT02045589	Advanced	VCN-01 + Nab-P + GEM	I	Active, not recruiting
NCT03481920	Advanced or locally advanced	PEGPH20 + Avelumab	I	Recruiting
NCT02734160	Metastatic	Galunisertib + Durvalumab	I	Recruiting

rand: randomized; pts: patients; GEM: gemcitabine; Nab-P: nab-paclitaxel; CY: Cyclophosphamide; SBRT: stereotactic body radiation therapy; CIS: chromosomal instability; HRD: homologous recombination repair deficiency; ➔: followed by. ^∗^PANC 10.05 pcDNA-1/GM-Neo and PANC 6.03 pcDNA-1 neo vaccine.
